# Impact of baseline renal function on the efficacy and safety of different Anticoagulants in Atrial Fibrillation Patients – A cohort study

**DOI:** 10.1186/s12959-022-00423-w

**Published:** 2022-10-13

**Authors:** Wei-Chieh Lee, Ting-Wei Liao, Hsiu-Yu Fang, Po-Jui Wu, Yen-Nan Fang, Huang-Chung Chen, Yu-Sheng Lin, Shang-Hung Chang, Ping-Yen Liu, Mien-Cheng Chen

**Affiliations:** 1 Institute of Clinical Medicine, College of Medicine, National Cheng Kung University, Tainan, Taiwan; 2 Division of Cardiology, Department of Internal Medicine, Chi Mei Medical Center, Tainan, Taiwan; 3 Division of Cardiology, Department of Internal Medicine, Kaohsiung Chang Gung Memorial Hospital, Chang Gung University College of Medicine, 123 Ta Pei Road, Niao Sung District, 83301 Kaohsiung City, Taiwan; 4 Center for Big Data Analytics and Statistics, Chang-Gung University and Hospital, Taipei, Taiwan; 5 Division of Cardiology, Chang Gung Memorial Hospital, Chiayi, Taiwan; 6 Division of Cardiology, Internal Medicine, College of Medicine, National Cheng Kung University Hospital, National Cheng Kung University, Tainan, Taiwan

**Keywords:** Atrial fibrillation, Direct oral anticoagulant, Renal impairment, Vitamin K antagonists, Safety

## Abstract

**Background:**

Vitamin K antagonists and different direct oral anticoagulants (DOACs) have different renal clearance rates. However, the impact of different stages of chronic renal impairment on the efficacy and safety of warfarin, dabigatran, rivaroxaban, apixaban, and edoxaban in atrial fibrillation (AF) patients remains unclear.

**Methods:**

This study enrolled AF patients from the Chang Gung Research Database. The study endpoints included thromboembolic events, major/fatal bleeding, gastrointestinal (GI) bleeding and intracranial hemorrhage (ICH). The risks of time to study endpoints between groups were compared using a Cox proportional hazards regression model with adjustment.

**Results:**

This study enrolled 3525 patients with moderate renal impairment (30 ≤ creatinine clearance (CrCl) < 60 mL/min), 2846 patients with mild renal impairment (60 ≤ CrCl < 90 mL/min) and 1153 patients with CrCl ≥ 90 mL/min. Over the 3.3 ± 0.9 years follow-up period, the cumulative thromboembolic events rates and the cumulative event rates of major/fatal bleeding and ICH did not differ among the warfarin and different DOAC groups at different stages of chronic renal impairment. The annual incidences of thromboembolic events, major/fatal bleeding, GI bleeding, and ICH were similar among the warfarin and different DOAC groups at different stages of renal impairment.

**Conclusion:**

There did not appear to be major differences in bleeding or thromboembolic risk compared to warfarin in AF patients across a range of degree of renal failure when appropriate dose reductions of the DOACs are made.

**Supplementary Information:**

The online version contains supplementary material available at 10.1186/s12959-022-00423-w.

## Background

Vitamin K antagonists and different direct oral anticoagulants (DOACs) have different renal clearance rates. Renal clearances of dabigatran, rivaroxaban, apixaban, and edoxaban are approximately 80%, 35%, 27%, and 50%, respectively. [1] However, increased exposure of rivaroxaban and apixaban might develop in patients with advanced renal impairment, even though they had less renal clearance compared to dabigatran and edoxaban. [2, 3] Randomized studies have shown that DOACs have comparable efficacy and better safety for stroke prevention in patients with non-valvular atrial fibrillation (AF) compared to warfarin. [4, 5, 6, 7] In the four major randomized studies of DOACs and AF, 17-21% of the enrolled patients had chronic kidney disease (CKD). [4, 5, 6, 7] In the real-world practice, the prevalence of mild-to-moderate renal impairment is approximately 60% in patients with AF, and up to 4% of AF patients have severe renal impairment. [8] Moreover, renal impairment is associated with an increased risk of thromboembolic events and major bleeding in patients with AF. [9, 10] Furthermore, proteinuria and reduced glomerular filtration rate are associated with higher rates of thromboembolism, independent of other stroke risk factors in patients with AF. [11] The possible mechanisms are related to the renin-angiotensin-aldosterone system activation and increase in inflammatory markers, fibrinogen and von Willebrand factor. [12] Therefore, management of AF patients concomitant with CKD is challenging for physicians in terms of stroke prevention and bleeding. According to the pharmacokinetics of different DOACs, the recommended dosage of different DOACs for AF patients with CKD ranged from 25 to 50% of recommended full dosage. [1] A recommendation for the use of dabigatran 110 mg twice daily is made in patients with creatinine clearance (CrCl) < 50 mL/min at high risk of bleeding. [1] Rivaroxaban (10 mg daily), apixaban (2.5 mg twice daily), and edoxaban (30 mg daily) (but not dabigatran) are approved for the use in patients with severe CKD (stage 4, i.e., a CrCl of 15–29 mL/min). [1] Moreover, apixaban 2.5-mg twice daily is recommended in patients with two or more of the following criteria: an age of at least 80 years, a body weight of no more than 60 kg, or a serum creatinine level of 1.5 mg per deciliter or more, edoxaban 30 mg daily for patients if any of the following characteristics is present, estimated CrCl of 30 to 50 mL/minute, a body weight of 60 kg or less, or the concomitant use of verapamil, quinidine, or potent P-glycoprotein inhibitors, and rivaroxaban 15 mg daily in patients with a CrCl of 30 to 49 mL/minute. Currently, one lower dosage is available for dabigatran, apixaban and edoxaban, and two lower dosages are available for rivaroxaban. However, the impact of baseline different stages of chronic renal impairment on the efficacy and safety of warfarin and different DOACs for stroke prevention in patients with AF remains unclear. Accordingly, we conducted this large retrospective cohort study with detailed laboratory data to explore the impact of different stages of chronic renal impairment on the efficacy and safety of warfarin and different DOACs in Taiwanese population with AF.

## Methods

### Patient population

This study enrolled patients with non-valvular AF from January 2001 to December 2017, and the medical history, detailed laboratory data and drug use of the patients were obtained from the Chang Gung Research Database (CGRD). The CGRD comprises four tertiary care medical centers and three major teaching hospitals with a total of 10,050 beds and is the largest healthcare system in Taiwan. [13]

The inclusion criteria were as follows: age ≥ 18 years with diagnosis of AF (International Classification of Diseases, Ninth Revision, Clinical Modification [ICD-9-CM] code 427.31 or Tenth Revision [ICD-10] codes I480, I481, I482, and I4891), and patients who were prescribed the same oral anticoagulant (warfarin or DOACs) for more than 1 year. All patients had been in stable CrCl for more than 90 days. All patients in the DOAC groups underwent dosage adjustment according to the dosing criteria of each DOAC, especially when renal function declined.

**Fig. 1:**
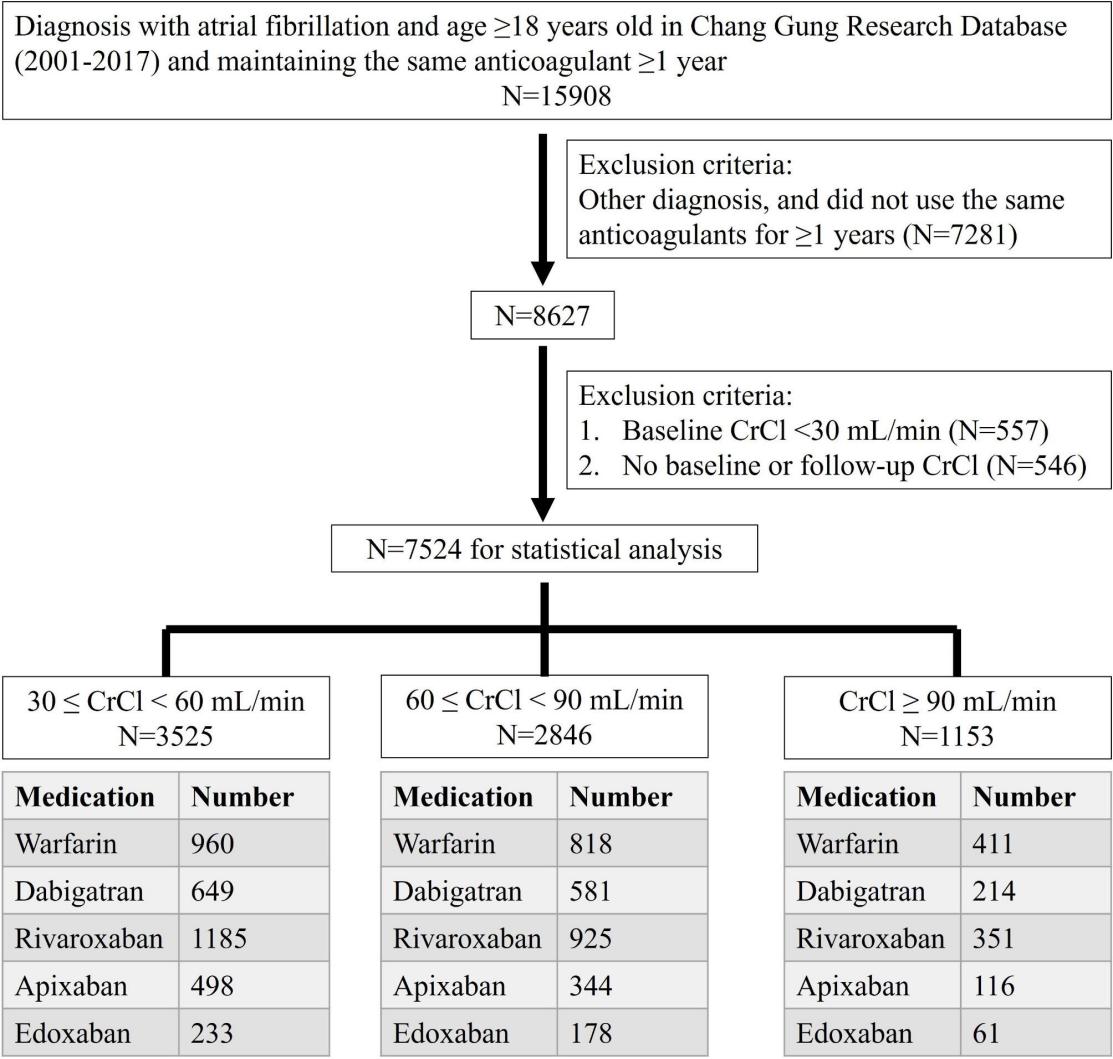
Flow chart of the study population N: number; CrCl: creatinine clearance

Patients who received oral anticoagulants for mechanical valves, rheumatic mitral stenosis, pulmonary embolism, venous thromboembolism, or preventive use after orthopedic surgery, patients with baseline CrCl < 30 mL/min and patients without baseline or follow-up renal function available were excluded. The flowchart of the study population is presented in Fig. 1.

Data on general demographics, comorbidities, baseline CrCl, medication use, bleeding events, including major/fatal bleeding, intracranial hemorrhage (ICH), and gastrointestinal (GI) bleeding, and thromboembolic events of patients in the warfarin and DOACs groups were obtained and analyzed. The study patients were also stratified into 3 renal function subgroups based on baseline CrCl, and the study endpoints were compared among the warfarin and DOACs groups.

### Definition

CrCl was estimated using the Cockcroft-Gault equation. Major and fatal bleeding events was defined as admission owing to ICH or bleeding in the critical organ or area, GI bleeding, or any bleeding requiring ≥ 2 units of packed red blood cell transfusion according to ISTH major bleeding criteria. [14] Thromboembolic events were defined and included stroke or vascular thromboembolic occlusion of an extremity or extracranial vital organ. [15] Inappropriate low dose of DOAC was defined as the reduced dosage was not adhered to the dosing adjustment criteria of each DOACs as recommended by guideline. [1]

### Study endpoint

The study endpoints included thromboembolic events, major and fatal bleeding events, GI bleeding, and ICH. We accepted patients as meeting a study endpoint (major and fatal bleeding or thromboembolic events) if they were categorized as such by the ICD discharge code.

## Statistical analyses

Data are presented as the mean ± standard deviation or numbers (percentages). The clinical characteristics of the two groups were compared using the independent samples t-test for continuous variables and the chi-squared test or Fisher’s exact test for categorical variables. The clinical characteristics of the four DOAC groups were compared using analysis of variance for continuous variables and the Kruskal-Wallis test for categorical variables. Significant difference was set at 0.05 level by Bonferroni multiple comparison procedure for each group. The risks of time to study endpoints between groups were compared using a Cox proportional hazards regression model, and the adjustment of age, sex, baseline CrCl, CHA2DS2-VASc score, HAS-BLED score, and the exclusion of inappropriate low dose of DOAC were performed. The risks, in terms of hazard ratios (HRs), of thromboembolic events, major/fatal bleeding, GI bleeding, and ICH were compared among the warfarin and different DOAC groups at different stages of chronic renal impairment. Kaplan-Meier curve analysis was performed with the log-rank test for thromboembolic events, major/fatal bleeding, GI bleeding, and ICH in groups during the 3-year follow-up period. Statistical significance was set at P < 0.05. All analyses were performed using SAS version 9.4 (SAS Institute. Inc., Cary, NC, USA).

## Results

### Baseline characteristics of the warfarin and DOAC groups in the whole study population

This study enrolled 7524 participants (Fig. 1), and the baseline clinical characteristics are listed in the Table 1. The DOAC groups were older and had higher prevalence of male gender, diabetes mellitus, hypertension, hyperlipidemia and vascular disease, a lower prevalence of heart failure, a lower baseline CrCl, a lower CHA2DS2-VASc score and a lower HAS-BLED score than the warfarin group. In warfarin group, the mean follow-up value of international normalized ratio (INR) was 2.14 ± 0.71.

**Table 1:** Baseline characteristics of whole study group

Variables	Warfarin	DOAC				*P* value (Warfarin vs. DOAC)	*P* value
		Dabigatran	Rivaroxaban	Apixaban	Edoxaban		(4 DOACs)
***Number***	2189	1444	2461	958	472		
***Gender (male)***	1175 (53.68)	900 (62.33)	1455 (59.12)	583 (60.86)	297 (62.92)	< 0.01	0.16
***Age (years)***	64.67 (11.94)	69.79 (9.93) ^a^	70.44 (9.69) ^a^	71.12 (9.94) ^a^	70.36 (10.04) ^b^	< 0.01	0.01
***Comorbidities***							
Type 2 DM (%)	418 (19.10)	333 (23.06)	602 (24.46)	240 (25.05)	114 (24.15)	< 0.01	0.68
Hypertension (%)	1014 (46.32)	843 (58.38) ^a^	1521 (61.80) ^a^	617 (64.41) ^b^	295 (62.50) ^a^	< 0.01	0.02
Hyperlipidemia (%)	483 (22.06)	413 (28.60)	716 (29.09)	320 (33.40)	144 (30.51)	< 0.01	0.05
Heart failure (%)	635 (29.01)	269 (18.63)	534 (21.70)	200 (20.88)	91 (19.28)	< 0.01	0.12
Prior stroke (%)	226 (10.32)	222 (15.37) ^a^	272 (11.05) ^b^	81 (8.46) ^b^	28 (5.93) ^c^	< 0.01	< 0.01
Vascular disease (%)	50 (2.28)	36 (2.49) ^a^	72 (2.93) ^a^	60 (6.26) ^b^	23 (4.87) ^b^	< 0.01	< 0.01
***Renal function***							
Serum Cr (mg/dL)	1.01 (0.33)	0.98 (0.27) ^a^	1.00 (0.29) ^a^	1.05 (0.35) ^b^	1.04 (0.31) ^b^	< 0.01	< 0.01
Baseline CrCl (mL/min)	68.55 (27.74)	67.73 (25.95) ^a^	65.15 (24.32) ^b^	62.5 (24.08) ^c^	63.63 (23.84) ^c^	< 0.01	< 0.01
***CHA2DS2-VASc score***	2.38 (1.53)	2.76 (1.50)	2.84 (1.51)	2.87 (1.44)	2.73 (1.52)	< 0.01	0.15
***HAS-BLED score***	1.36 (1.05)	1.65 (0.92) ^a^	1.72 (0.93) ^a^	1.78 (0.92) ^a^	1.70 (0.97) ^b^	< 0.01	< 0.01

Data are expressed as mean (standard deviation) or median (interquartile range) or as a number (percentage)

Different letters (a, b, c) associated with different groups indicate significant difference (at 0.05 level) by Bonferroni multiple comparison procedure

### Dose distribution of each DOAC

In the dabigatran group, 83% of the patients received 110 mg twice daily, and the percentage of patients received dabigatran 110 mg twice daily increased from 68.22% in patients with CrCl ≥ 90 mL/min to 90.14% in patients with moderate renal impairment (30 ≤ CrCl < 60 mL/min) (Supplemental Fig. 1 ). In the rivaroxaban group, 34.0% of the patients received 10 mg daily, and 2.0% received rivaroxaban below 10 mg daily (Supplemental Fig. 2). The percentage of patients received rivaroxaban below 15 mg daily increased from 18.52% in patients with CrCl ≥ 90 mL/min to 45.65% in patients with moderate renal impairment (30 ≤ CrCl < 60 mL/min) (Supplemental Fig. 2). In the apixaban group, more than half of the patients received apixaban 2.5 mg twice daily, and the percentage of patients received apixaban 2.5 mg twice daily increased from 16.38% in patients with CrCl ≥ 90 mL/min to 68.88% in patients with moderate renal impairment (30 ≤ CrCl < 60 mL/min) (Supplemental Fig. 3). In the edoxaban group, 65% of patients received edoxaban below 60 mg daily, and the percentage of patients received edoxaban below 60 mg daily increased from 40.98% in patients with CrCl ≥ 90 mL/min to 79.4% in patients with moderate renal impairment (30 ≤ CrCl < 60 mL/min) (Supplemental Fig. 4).

### Baseline characteristics of the warfarin and DOAC groups in patients with moderate renal impairment (30 ≤ CrCl < 60 mL/min), mild renal impairment (60 ≤ CrCl < 90 mL/min), and renal function of CrCl ≥ 90 mL/min

In patients with moderate renal impairment (30 ≤ CrCl < 60 mL/min), the DOAC groups were older and had higher prevalence of male gender, hypertension and hyperlipidemia, a lower prevalence of heart failure, a lower CHA2DS2-VASc score and a lower HAS-BLED score than the warfarin group (Supplemental Table 1). The highest prevalence of prior stroke was noted in the dabigatran group and the highest prevalence of vascular disease was noted in the apixaban group. The dabigatran and rivaroxaban groups had a lower serum Cr level than the warfarin, apixaban and edoxaban groups. In the warfarin group with moderate renal impairment (30 ≤ CrCl < 60 mL/min), the mean follow-up value of INR was 2.16 ± 0.91.

In patients with mild renal impairment (60 ≤ CrCl < 90 mL/min), the DOAC groups were older and had higher prevalence of male gender, hypertension, hyperlipidemia and vascular disease, a lower prevalence of heart failure, a lower baseline CrCL, a higher CHA2DS2-VASc score and a higher HAS-BLED score than the warfarin group (Supplemental Table 2). The highest prevalence of prior stroke was noted in the dabigatran group and the highest prevalence of vascular disease was noted in the edoxaban group. In the warfarin group with mild renal impairment (60 ≤ CrCl < 90 mL/min), the mean follow-up value of INR was 2.15 ± 0.71.

In patients with renal function (CrCl ≥ 90 mL/min), the DOAC groups were older and had higher prevalence of male gender, hypertension, and prior stroke, a higher CHA2DS2-VASc score and a higher HAS-BLED score than the warfarin group (Supplemental Table 3). The highest prevalence of prior stroke was noted in the dabigatran group. Serum Cr and baseline CrCl levels were similar across the warfarin and different DOAC groups. In the warfarin group with renal function (CrCl ≥ 90 mL/min), the mean follow-up value of INR was 2.09 ± 0.41.

### Annual incidences of clinical outcomes of patients at different stages of renal impairment

In patients with moderate renal impairment (30 ≤ CrCl < 60 mL/min), the annual incidences of thromboembolic events, major/fatal bleeding, GI bleeding, and ICH were similar among the warfarin and different DOAC groups (Table 2). In patients with mild renal impairment (60 ≤ CrCl < 90 mL/min), the annual incidences of thromboembolic events, major/fatal bleeding, GI bleeding, and ICH were similar among the warfarin and different DOAC groups. In patients with renal function of CrCl ≥ 90 mL/min, the annual incidences of thromboembolic events, major/fatal bleeding, GI bleeding, and ICH were similar among the warfarin and different DOAC groups.

**Table 2:** Annual incidences of clinical outcomes of patients with renal function of 30 ≤ CrCl < 60 mL/min, 60 ≤ CrCl < 90 mL/min and CrCl ≥ 90 mL/min

Variables	Warfarin	DOAC				*P* value (Warfarin vs. DOAC)	*P* value
		Dabigatran	Rivaroxaban	Apixaban	Edoxaban		(4 DOACs)
***30 ≤ CrCl < 60 mL/min***							
Thromboembolic events (%)	1.22	0.72	1.01	1.41	1.29	0.30	0.22
Major and fatal bleeding (%)	0.94	0.56	0.76	0.74	0.43	0.51	0.69
GI bleeding (%)	1.46	0.87	1.29	1.67	0.86	0.18	0.14
Intracranial hemorrhage (%)	0.63	0.21	0.48	0.33	0.29	0.22	0.42
***60 ≤ CrCl < 90 mL/min***							
Thromboembolic events (%)	0.57	0.57	0.90	0.78	0.75	0.62	0.68
Major and fatal bleeding (%)	0.37	0.34	0.25	0.29	0.19	0.92	0.93
GI bleeding (%)	0.53	0.52	0.58	0.87	0.56	0.78	0.68
Intracranial hemorrhage (%)	0.49	0.34	0.36	0.10	0.19	0.45	0.54
***CrCl ≥ 90 mL/min***							
Thromboembolic events (%)	0.81	1.40	0.38	0.86	1.09	0.24	0.09
Major and fatal bleeding (%)	0.32	0	0.19	0.29	0	0.60	0.45
GI bleeding (%)	0.57	0.16	0.66	0.57	0	0.52	0.41
Intracranial hemorrhage (%)	0.57	0	0.19	0.29	0	0.19	0.45

Abbreviation: CrCl: creatinine clearance; DOAC: direct oral anticoagulant; GI: gastrointestinal

### Kaplan-Meier curve and hazard ratios for clinical outcomes among different anticoagulants

**Fig. 2:**
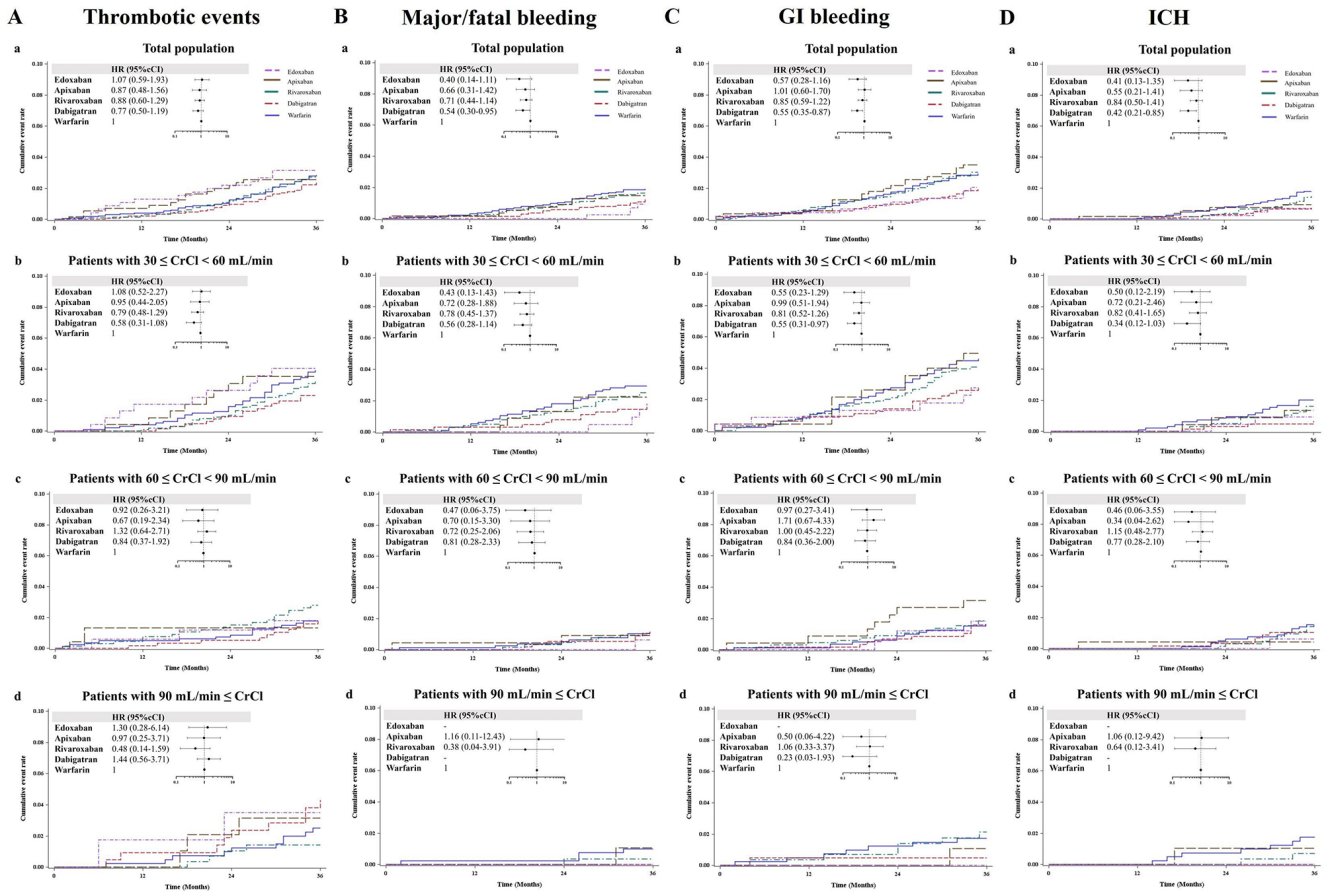
In the Kaplan-Meier Curve and Cox proportional hazards regression model, hazard ratios of thromboembolic events, major/fatal bleeding, GI bleeding, and ICH were compared among the warfarin and different DOACs groups at different stages of renal function after adjustment of age, sex, CrCl, CHA2DS2-VASc score, HAS-BLED score, and the exclusion inappropriate low dosage of DOACs A: Thromboembolic events B: Major/fatal bleeding C: GI bleeding D: ICH DOAC: direct oral anticoagulants; GI: gastrointestinal; ICH: intracranial hemorrhage; CrCl: creatinine clearance; HR: hazard ratio; CI: confidence interval

In a Cox proportional hazards regression model, the HRs of thromboembolic events, major/fatal bleeding, GI bleeding, and ICH were analyzed among the warfarin and different DOAC groups at different stages of chronic renal impairment after adjustment of age, sex, baseline CrCl, CHA2DS2-VASc score, HAS-BLED score, and the exclusion of inappropriate low dosage of DOAC and are summarized in Fig. 2. Over the 3.3 ± 0.9 years follow-up period, the cumulative thromboembolic event rate did not differ among the warfarin and different DOAC groups in the whole study population and at different stages of chronic renal impairment. In the whole study population, the dabigatran group had lower cumulative event rates of major/fatal bleeding (HR = 0.54; 95% confidence interval [CI], 0.30–0.95) (Fig. 2Ba), GI bleeding (HR = 0.55; 95% CI, 0.35–0.87) (Fig. 2Ca) and ICH (HR = 0.42; 95% CI, 0.21–0.85) (Fig. 2Da) than the warfarin group. In patients with moderate renal impairment, the dabigatran group had a lower cumulative event rate of GI bleeding (HR = 0.55; 95% CI, 0.31–0.97) (Fig. 2Cb) than the warfarin group. However, in patients without moderate renal impairment, the dabigatran group did not differ in the cumulative event rate of GI bleeding compared to the warfarin group. Interestingly, the dabigatran group did not differ in the cumulative event rates of major/fatal bleeding and ICH compared to the warfarin group in patients at different stages of chronic renal impairment. The edoxaban, apixaban and rivaroxaban groups did not differ in the cumulative event rates of major/fatal bleeding, GI bleeding and ICH compared to the warfarin group in the whole study population and at different stages of chronic renal impairment.

## Discussion

In this large retrospective cohort study, we found that the cumulative thromboembolic event rate did not differ among the warfarin and different DOAC groups in the whole study population and in patients at different stages of chronic renal impairment over the 3.3 ± 0.9 years follow-up period. The dabigatran group did not differ in the cumulative event rate of GI bleeding in patients without moderate renal impairment compared to the warfarin group. The dabigatran group did not differ in the cumulative event rates of major/fatal bleeding and ICH compared to the warfarin group in patients at different stages of chronic renal impairment. The apixaban, edoxaban and rivaroxaban groups did not differ in the cumulative event rates of major/fatal bleeding, GI bleeding and ICH compared to the warfarin group in patients at different stages of chronic renal impairment. The annual incidences of thromboembolic events, major/fatal bleeding, GI bleeding, and ICH were similar among the warfarin and different DOAC groups at different stages of renal impairment.

Different DOACs have different renal clearance rates. In the ENGAGE AF-TIMI 48 study, edoxaban yielded a higher incidence of thromboembolic events in patients with supranormal renal function (CrCl ≥ 90 mL/min) than warfarin. [7] Moreover, a case report showed reduced efficacy in AF patient with protein S deficiency and supranormal renal function under treatment with standard dose edoxaban. [16] In contrast, a study using Korean registry database showed that dabigatran, apixaban, edoxaban and rivaroxaban had better effectiveness and safety than warfarin in AF patients with supranormal renal function (CrCl > 95 mL/min). [17] Therefore, the effectiveness and safety of DOACs compared to warfarin in patients with CrCl ≥ 90 mL/min remain inconclusive and the efficacy and safety of DOACs in AF patients with different ethnicities and different renal function remain unclear. Moreover, renal impairment is associated with an increased risk of thromboembolic events and major bleeding in patients with AF. [9, 10 A] previous study using a US administrative claims database reported that apixaban was associated with a lower risk of major bleeding compared to warfarin in AF patients with mild-to-moderate renal impairment. [18] In this study of Taiwanese patients with AF, we showed that the cumulative thromboembolic event rates did not differ among the warfarin and different DOAC groups in patients at different stages of chronic renal impairment. The dabigatran group did not differ in the cumulative event rate of GI bleeding in patients without moderate renal impairment compared to the warfarin group. The apixaban, edoxaban and rivaroxaban groups did not differ in the cumulative event rate of GI bleeding compared to the warfarin group in patients at different stages of chronic renal impairment.

## Study limitations

This study had several limitations. First, the study design was retrospective in nature. Second, this was not a randomized study and had potential selective bias. Even through adjustment of age, sex, baseline CrCl, CHA2DS2-VASc score, HAS-BLED score, and the exclusion of inappropriate low dose of DOAC, there were unmeasured confounders that might have impact on clinical outcomes. Third, the sample size was small in the subgroup analyses, and large CI reflected a high degree of evidence uncertainty. Fourth, only baseline CrCls were used for stratification. Fifth, patients with baseline CrCl < 30 mL/min were excluded in this cohort study. Sixth, the incidence of non-major bleeding events could not be available in the medical record in this cohort study. Seventh, the time in therapeutic range for the warfarin group at different renal stages could not be available in this cohort study, but we provided the mean follow-up value of INR in the warfarin group at different stages of renal impairment. Finally, the ICD-9 M and ICD-10 M codes relied on the physician’s choice in clinical practice. Adequately powered trials should be conducted to determine optimal treatment strategies for patients with different renal functions, especially for patients with advanced or moderate renal impairment. Nevertheless, this study provides valuable real-world evidence to guide clinical decisions to use DOACs for AF patients at different stages of chronic renal impairment.

## Conclusion

There did not appear to be major differences in bleeding or thromboembolic risk compared to warfarin in AF patients across a range of degree of renal failure when appropriate dose reductions of the DOACs are made. Further large randomized studies are warranted to validate our findings.

## Electronic supplementary material

Below is the link to the electronic supplementary material.

Supplementary Material 1

Supplementary Material 2

Supplementary Material 3

Supplementary Material 4

## Data Availability

The study data will be available from the corresponding author upon reasonable request. This study was based in part on data from the CGRD provided by the Chang Gung Memorial Hospital. The interpretation and conclusions contained herein do not represent the position of the Chang Gung Memorial Hospital.

## Abbreviations

DOAC: direct oral anticoagulant.
DM: diabetes mellitus.
Cr: creatinine.
CrCl: creatinine clearance.
