# Supplementary material for: Impact of baseline renal function on the efficacy and safety of different Anticoagulants in Atrial Fibrillation Patients – A cohort study
Source: Thromb J. 2022 Oct 13;20:64. doi: 10.1186/s12959-022-00423-w (PMC9559011; doi:10.1186/s12959-022-00423-w)

Supplemental figure legends

Supplemental Figure 1.

Dose distribution in the dabigatran group, 83% of the patients received 110 mg twice a day. The percentage of patients received dabigatran 110 mg twice daily increased from 68.22% in patients with CrCl ≥ 90 mL/min to 90.14% in patients with moderate renal impairment (30 ≤ CrCl < 60 mL/min).

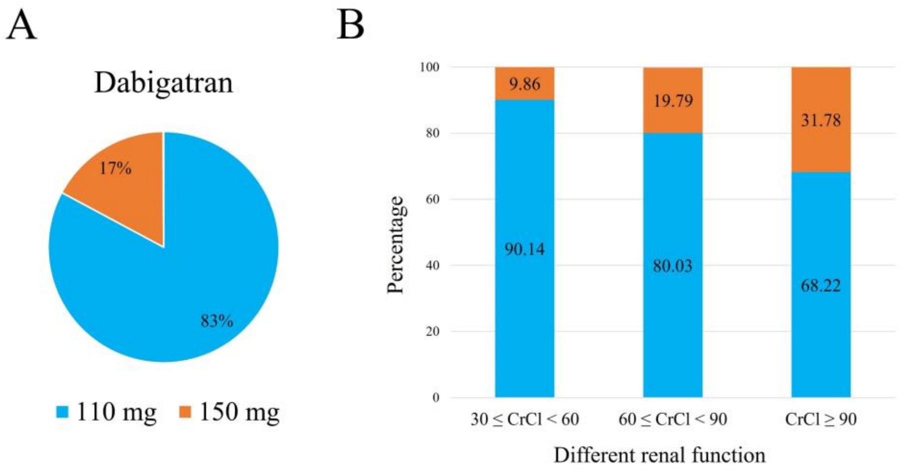

Supplemental Figure 2.

Dose distribution in the rivaroxaban group, 34.0% of the patients received 10 mg daily, and 2.0% received rivaroxaban below 10 mg daily.

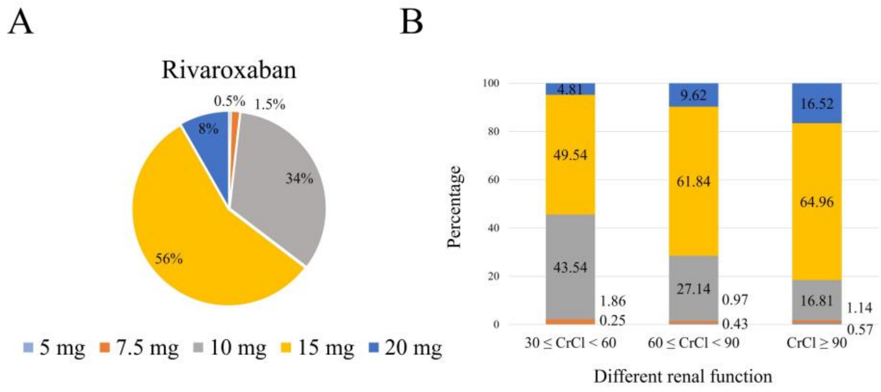

Supplemental Figure 3.

Dose distribution in the apixaban group, 51%f of the patients received apixaban 2.5 mg twice daily.

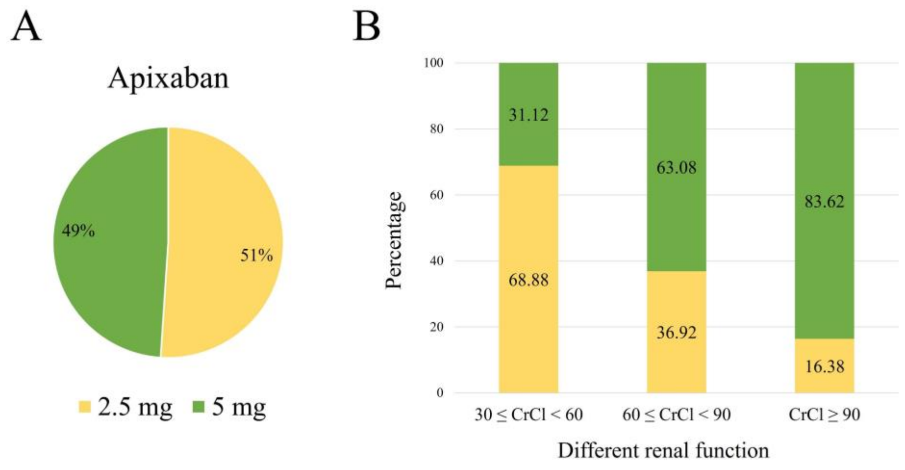

Supplemental Figure 4.

Dose distribution in the edoxaban group, 65% of patients received edoxaban below 60 mg daily.

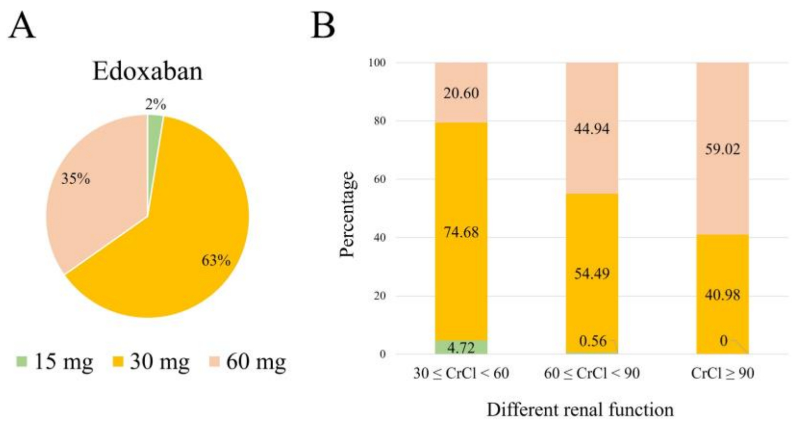

Supplement: Supplementary file 1 — Supplementary Material 1 [file 12959_2022_423_MOESM1_ESM.pdf]
