# Supplementary material for: Impact of baseline renal function on the efficacy and safety of different Anticoagulants in Atrial Fibrillation Patients – A cohort study
Source: Thromb J. 2022 Oct 13;20:64. doi: 10.1186/s12959-022-00423-w (PMC9559011; doi:10.1186/s12959-022-00423-w)
Supplement: Supplementary file 2 — Supplementary Material 2 [file 12959_2022_423_MOESM2_ESM.docx]

Supplemental Table 1.

Baseline characteristics of atrial fibrillation patients with renal function of 30 ≤ CrCl < 60 mL/min

| Variables | Warfarin | DOAC | | | | *P* value  (Warfarin vs. DOAC) | *P* value |
| --- | --- | --- | --- | --- | --- | --- | --- |
|  |  | Dabigatran | Rivaroxaban | Apixaban | Edoxaban |  | (4 DOACs) |
| ***Number*** | 960 | 649 | 1185 | 498 | 233 |  |  |
| ***Gender (male)*** | 464 (48.33) | 359 (55.32) | 620 (52.32) | 280 (56.22) | 137 (58.80) | <0.01 | 0.19 |
| ***Age (years)*** | 71.55 (9.52) | 75.44 (7.70) | 75.21 (7.83) | 75.66 (8.43) | 75.80 (7.89) | <0.01 | 0.60 |
| ***Comorbidities*** |  |  |  |  |  |  |  |
| Type 2 DM (%) | 207 (21.56) | 163 (25.12) | 311 (26.24) | 134 (26.91) | 59 (25.32) | 0.09 | 0.90 |
| Hypertension (%) | 513 (53.44) | 400 (61.63) | 773 (65.23) | 343 (68.88) | 156 (66.95) | <0.01 | 0.07 |
| Hyperlipidemia (%) | 229 (23.85) | 187 (28.81) | 376 (31.73) | 168 (33.73) | 75 (32.19) | <0.01 | 0.33 |
| Heart failure (%) | 298 (31.04) | 114 (17.57) ^a^ | 279 (23.54) ^b^ | 110 (22.09) ^b^ | 48 (20.60) ^c^ | <0.01 | 0.03 |
| Prior stroke (%) | 116 (12.08) | 90 (13.87) ^a^ | 140 (11.81) ^a^ | 38 (7.63) ^c^ | 19 (8.15) ^c^ | 0.01 | <0.01 |
| Vascular disease (%) | 36 (3.75) | 18 (2.77) ^a^ | 42 (3.54) ^a^ | 36 (7.23) ^b^ | 7 (3.00) ^a^ | <0.01 | <0.01 |
| ***Renal function*** |  |  |  |  |  |  |  |
| Serum Cr (mg/dL) | 1.19 (0.35) | 1.10 (0.27) ^a^ | 1.13 (0.30) ^a^ | 1.20 (0.37) ^b^ | 1.19 (0.32) ^b^ | <0.01 | <0.01 |
| Baseline CrCl (mL/min) | 45.68 (8.31) | 47.81 (7.61) ^a^ | 46.34 (8.15) ^b^ | 44.98 (8.79) ^b, c^ | 44.94 (9.08) ^c^ | <0.01 | <0.01 |
| ***CHA2DS2-VASc score*** | 3.06 (1.51) | 3.29 (1.43) | 3.36 (1.38) | 3.33 (1.21) | 3.30 (1.39) | <0.01 | 0.73 |
| ***HAS-BLED score*** | 1.99 (0.93) | 2.07 (0.84) ^a^ | 2.15 (0.81) ^a, b^ | 2.21 (0.78) ^b^ | 2.21 (0.86) ^b^ | <0.01 | 0.02 |

Data are expressed as mean (standard deviation) or median (interquartile range) or as a number (percentage).

Different letters (a, b, c) associated with different groups indicate significant difference (at 0.05 level) by Bonferroni multiple comparison procedure.

Abbreviation: CrCl: creatinine clearance; DOAC: direct oral anticoagulant; DM: diabetes mellitus; Cr: creatinine.
