# Supplementary material for: Impact of baseline renal function on the efficacy and safety of different Anticoagulants in Atrial Fibrillation Patients – A cohort study
Source: Thromb J. 2022 Oct 13;20:64. doi: 10.1186/s12959-022-00423-w (PMC9559011; doi:10.1186/s12959-022-00423-w)
Supplement: Supplementary file 3 — Supplementary Material 3 [file 12959_2022_423_MOESM3_ESM.docx]

Supplemental Table 2.

Baseline characteristics of atrial fibrillation patients with renal function of 60 ≤ CrCl < 90 mL/min

| Variables | Warfarin | DOAC | | | | *P* value  (Warfarin vs. DOAC) | *P* value |
| --- | --- | --- | --- | --- | --- | --- | --- |
|  |  | Dabigatran | Rivaroxaban | Apixaban | Edoxaban |  | (4 DOACs) |
| ***Number*** | 818 | 581 | 925 | 344 | 178 |  |  |
| ***Gender (male)*** | 476 (58.19) | 387 (66.61) | 602 (65.08) | 219 (63.66) | 113 (63.48) | 0.01 | 0.78 |
| ***Age (years)*** | 62.16 (9.96) | 67.1 (8.27) | 67.88 (8.17) | 68.03 (8.27) | 67.29 (8.16) | <0.01 | 0.22 |
| ***Comorbidities*** |  |  |  |  |  |  |  |
| Type 2 DM (%) | 139 (16.99) | 122 (21.00) | 208 (22.49) | 79 (22.97) | 40 (22.47) | 0.04 | 0.88 |
| Hypertension (%) | 339 (41.44) | 329 (56.63) | 562 (60.76) | 202 (58.72) | 105 (58.99) | <0.01 | 0.47 |
| Hyperlipidemia (%) | 177 (21.64) | 180 (30.98) | 253 (27.35) | 116 (33.72) | 58 (32.58) | <0.01 | 0.10 |
| Heart failure (%) | 220 (26.89) | 106 (18.24) | 180 (19.46) | 64 (18.60) | 32 (17.98) | <0.01 | 0.93 |
| Prior stroke (%) | 85 (10.39) | 92 (15.83) ^a^ | 103 (11.14) ^b^ | 28 (8.14) ^b^ | 5 (2.81) ^c^ | <0.01 | <0.01 |
| Vascular disease (%) | 8 (0.98) | 16 (2.75) ^a^ | 21 (2.27) ^a^ | 19 (5.52) ^b^ | 14 (7.87) ^c^ | <0.01 | <0.01 |
| ***Renal function*** |  |  |  |  |  |  |  |
| Serum Cr (mg/dL) | 0.92 (0.23) | 0.93 (0.22) | 0.92 (0.21) | 0.92 (0.24) | 0.92 (0.22) | 0.97 | 0.99 |
| Baseline CrCl (mL/min) | 73.18 (8.63) | 73.16 (8.39) | 72.28 (8.12) | 71.84 (8.51) | 72.69 (8.73) | 0.04 | 0.09 |
| ***CHA2DS2-VASc score*** | 2.01 (1.40) | 2.45 (1.42) | 2.51 (1.47) | 2.48 (1.45) | 2.35 (1.42) | <0.01 | 0.58 |
| ***HAS-BLED score*** | 1.00 (0.87) | 1.40 (0.82) | 1.44 (0.80) | 1.40 (0.80) | 1.33 (0.79) | <0.01 | 0.39 |

Data are expressed as mean (standard deviation) or median (interquartile range) or as a number (percentage).

Different letters (a, b, c) associated with different groups indicate significant difference (at 0.05 level) by Bonferroni multiple comparison procedure.

Abbreviation: CrCl: creatinine clearance; DOAC: direct oral anticoagulant; DM: diabetes mellitus; Cr: creatinine.
