# Supplementary material for: Impact of baseline renal function on the efficacy and safety of different Anticoagulants in Atrial Fibrillation Patients – A cohort study
Source: Thromb J. 2022 Oct 13;20:64. doi: 10.1186/s12959-022-00423-w (PMC9559011; doi:10.1186/s12959-022-00423-w)
Supplement: Supplementary file 4 — Supplementary Material 4 [file 12959_2022_423_MOESM4_ESM.docx]

Supplemental Table 3.

Baseline characteristics of atrial fibrillation patients with renal function of CrCl ≥ 90 mL/min

| Variables | Warfarin | DOAC | | | | *P* value  (Warfarin vs. DOAC) | *P* value |
| --- | --- | --- | --- | --- | --- | --- | --- |
|  |  | Dabigatran | Rivaroxaban | Apixaban | Edoxaban |  | (4 DOACs) |
| ***Number*** | 411 | 214 | 351 | 116 | 61 |  |  |
| ***Gender (male)*** | 235 (57.18) | 154 (71.96) | 233 (66.38) | 84 (72.41) | 47 (77.05) | <0.01 | 0.23 |
| ***Age (years)*** | 53.57 (10.21) | 59.95 (9.31) | 61.09 (9.71) | 60.77 (9.26) | 58.59 (8.36) | <0.01 | 0.19 |
| ***Comorbidities*** |  |  |  |  |  |  |  |
| Type 2 DM (%) | 72 (17.52) | 48 (22.43) | 83 (23.65) | 27 (23.28) | 15 (24.59) | 0.24 | 0.98 |
| Hypertension (%) | 162 (39.42) | 114 (53.27) | 186 (52.99) | 72 (62.07) | 34 (55.74) | <0.01 | 0.37 |
| Hyperlipidemia (%) | 77 (18.73) | 46 (21.50) | 87 (24.79) | 36 (31.03) | 11 (18.03) | 0.04 | 0.16 |
| Heart failure (%) | 117 (28.47) | 49 (22.90) | 75 (21.37) | 26 (22.41) | 11 (18.03) | 0.12 | 0.87 |
| Prior stroke (%) | 25 (6.08) | 40 (18.69) ^a^ | 29 (8.26) ^b^ | 15 (12.93) ^b^ | 4 (6.56) ^b^ | <0.01 | <0.01 |
| Vascular disease (%) | 6 (1.46) | 2 (0.93) | 9 (2.56) | 5 (4.31) | 2 (3.28) | 0.21 | 0.27 |
| ***Renal function*** |  |  |  |  |  |  |  |
| Serum Cr (mg/dL) | 0.76 (0.21) | 0.76 (0.20) | 0.76 (0.20) | 0.78 (0.18) | 0.83 (0.18) | 0.08 | 0.05 |
| Baseline CrCl (mL/min) | 112.72 (23.97) | 113.39 (28.49) | 109.91 (20.45) | 109.99 (20.28) | 108.57 (16.23) | 0.23 | 0.26 |
| ***CHA2DS2-VASc score*** | 1.55 (1.13) | 2.01 (1.38) | 1.97 (1.41) | 2.05 (1.57) | 1.64 (1.34) | <0.01 | 0.28 |
| ***HAS-BLED score*** | 0.58 (0.72) | 1.02 (0.81) | 0.99 (0.83) | 1.09 (0.88) | 0.84 (0.76) | <0.01 | 0.28 |

Data are expressed as mean (standard deviation) or median (interquartile range) or as a number (percentage).

Different letters (a, b) associated with different groups indicate significant difference (at 0.05 level) by Bonferroni multiple comparison procedure.

Abbreviation: CrCl: creatinine clearance; DOAC: direct oral anticoagulant; DM: diabetes mellitus; Cr: creatinine.
